# Comparison of Serological Assays for the Detection of SARS-CoV-2 Antibodies

**DOI:** 10.3390/v13040713

**Published:** 2021-04-20

**Authors:** Joe James, Shelley Rhodes, Craig S. Ross, Paul Skinner, Samuel P. Smith, Rebecca Shipley, Caroline J. Warren, Hooman Goharriz, Lorraine M. McElhinney, Nigel Temperton, Edward Wright, Anthony R. Fooks, Tristan W. Clark, Sharon M. Brookes, Ian H. Brown, Ashley C. Banyard

**Affiliations:** 1 Animal and Plant Health Agency (APHA), Weybridge, Surrey KT15 3NB, UK; shelley.rhodes@apha.gov.uk (S.R.); craig.ross@apha.gov.uk (C.S.R.); paul.skinner@apha.gov.uk (P.S.); sasmith@sgul.ac.uk (S.P.S.); rebecca.shipley@apha.gov.uk (R.S.); caroline.warren@apha.gov.uk (C.J.W.); hooman.goharriz@apha.gov.uk (H.G.); lorraaine.mcelhinney@apha.gov.uk (L.M.M.); tony.fooks@apha.gov.uk (A.R.F.); sharon.brookes@apha.gov.uk (S.M.B.); ian.brown@apha.gov.uk (I.H.B.); 2 Institute for Infection and Immunity, St. George’s Hospital Medical School, University of London, London SW17 0RE, UK; 3 School of Life Sciences, University of Sussex, Falmer, Brighton BN1 9QG, UK; ew323@sussex.ac.uk; 4 Viral Pseudotype Unit, Medway School of Pharmacy, University of Kent and Greenwich, Chatham, Kent ME4 4TB, UK; n.temperton@greenwich.ac.uk; 5 School of Clinical and Experimental Sciences, University of Southampton, Southampton General Hospital, Tremona Road, Southampton SO16 6YD, UK; Tristan.clark@uhs.nhs.uk; 6 Department of Infection, University Hospital Southampton NHS Foundation Trust, Southampton SO16 6YD, UK

**Keywords:** coronavirus, COVID-19, SARS-CoV-2, spike glycoproteins, ELISA, IgG, neutralization, cross-reactivity, convalescent plasma, pseudotype neutralisation

## Abstract

SARS-CoV-2 virus was first detected in late 2019 and circulated globally, causing COVID-19, which is characterised by sub-clinical to severe disease in humans. Here, we investigate the serological antibody responses to SARS-CoV-2 infection during acute and convalescent infection using a cohort of (i) COVID-19 patients admitted to hospital, (ii) healthy individuals who had experienced ‘COVID-19 like-illness’, and (iii) a cohort of healthy individuals prior to the emergence of SARS-CoV-2. We compare SARS-CoV-2 specific antibody detection rates from four different serological methods, virus neutralisation test (VNT), ID Screen^®^ SARS-CoV-2-N IgG ELISA, Whole Antigen ELISA, and lentivirus-based SARS-CoV-2 pseudotype virus neutralisation tests (pVNT). All methods were able to detect prior infection with COVID-19, albeit with different relative sensitivities. The VNT and SARS-CoV-2-N ELISA methods showed a strong correlation yet provided increased detection rates when used in combination. A pVNT correlated strongly with SARS-CoV-2 VNT and was able to effectively discriminate SARS-CoV-2 antibody positive and negative serum with the same efficiency as the VNT. Moreover, the pVNT was performed with the same level of discrimination across multiple separate institutions. Therefore, the pVNT is a sensitive, specific, and reproducible lower biosafety level alternative to VNT for detecting SARS-CoV-2 antibodies for diagnostic and research applications. Our data illustrate the potential utility of applying VNT or pVNT and ELISA antibody tests in parallel to enhance the sensitivity of exposure to infection.

## 1. Introduction

Severe acute respiratory syndrome coronavirus 2 (SARS-CoV-2) was first linked to human disease following an outbreak characterised by respiratory distress in Wuhan, China in late 2019. The causative agent was subsequently isolated and characterised from infected humans [1] and was defined as a species of coronavirus; this virus was later classified as a betacoronavirus and termed SARS-CoV-2 [2]. This novel coronavirus, SARS-CoV-2, is classified alongside six other coronaviruses known to infect humans. Of these, SARS-CoV, MERS-CoV, and SARS-CoV-2 are associated with human disease, whilst HKU1, NL63, OC43, and 229E are more often associated with asymptomatic or mild clinical progression [1]. From a clinical perspective, SARS-CoV-2 infection can lead to the development of coronavirus disease 2019 (COVID-19), which was initially characterised, for example, by fever, cough, and anosmia (loss of sense of smell) with resultant ageusia (a loss of sense of taste) [3,4]. Clinical manifestations can vary significantly between affected individuals, with no pathognomonic manifestations currently defined. COVID-19 manifests clinically as asymptomatic (present in 30–40% of infected individuals), mild (present in ≈80% of symptomatic cases), and severe disease (present in ≈20% of symptomatic cases), with case fatality rates of between 0.4% and 11% [5]. In severe cases, mortality is generally attributed to respiratory failure, pneumonia, systemic shock, or multi-organ dysfunction [4]. Since its emergence, SARS-CoV-2 has demonstrated an ability to spread rapidly from human to human, primarily through direct or indirect contact with respiratory secretions and via inhalation of respiratory droplets and aerosols [6]. The World Health Organization declared the outbreak a Public Health Emergency of International Concern on 12 January 2020 and a global pandemic on 11 March 2020 [7]. As of 27 February 2021, there have been more than 113 million cases of COVID-19 globally with 2.5 million deaths attributed to COVID-19 (https://coronavirus.jhu.edu/map.html (accessed on 20 March 2021)) [8].

SARS-CoV-2, is a positive-sense, single-stranded RNA (+ssRNA) virus, with a single linear RNA segment as its genome [1]. SARS-CoV-2 virions are 50–200 nm in diameter and consist of four main structural proteins, including the spike (S) glycoprotein (the major immunogenic and antigenic virion component), the small envelope (E) glycoprotein, the membrane (M) glycoprotein, and the nucleocapsid (N) protein [1] alongside several accessory proteins. The S glycoprotein forms a homotrimer and protrudes from the surface of the SARS-CoV-2 virion and infected cells, and it is thought to preferentially utilise the angiotensin-converting enzyme 2 (ACE-2) receptor on host cells, facilitating internalisation and infection. The S glycoprotein is also the major antigenic component of the SARS-CoV-2 virus [9].

Tracking infections and hospitalisations have demonstrated a cyclic pattern of increasing and decreasing clinical prevalence, reflecting regional and national efforts to reduce transmission [10]. Alongside efforts to diagnose and track infection, interest has gathered in assessing immunological responses post infection [11]. The generation of novel vaccines has further stimulated the evaluation of responses to both infection and vaccination. As such, data regarding serological responses to infection, and any potentially protective outcome of having been infected, either with or without the development of clinical disease, have increased [11]. Indeed, serological tests have been widely heralded as a potential key assays in any exit strategy from community lockdowns across the globe. Several serological diagnostic technologies that assess specific immunity to pathogens have been developed for SARS-CoV-2 including virus neutralisation tests (VNTs) [12], viral pseudotype neutralisation tests (pVNTs) [13,14,15], and enzyme-linked immunosorbent assays (ELISAs) [16,17,18,19,20].

VNTs are often considered as the ‘gold standard’ for serological detection, as the results demonstrate inactivation of infectious virus, and as such, VNTs represent a strong correlative indicator of protection from disease. Outputs are generated as a relative titre, or dilution of serum, that inhibits virus-mediated cell death of a cultured mammalian cell line [21] and often rely on the availability of virus isolate that cause cytopathic effect (CPE) [22]. The level of neutralisation can be dependent on a variety of factors, including the cell type used in the assay, the species from which the blood sample derives, and the isolate of the virus being used in the test [21]. In the UK, the classical virus neutralisation test for SARS-CoV-requires the use of containment level 3 (CL3) facilities [12]. However, there has been interest in producing lower containment level alternatives to measure neutralising antibodies to provide faster, more practical diagnostic assays. Recently, surrogate virus neutralisation tests (sVNTs) have been described that use the principle of an ELISA to measure the neutralisation capacity of anti-SARS-CoV-2 antibodies directed against the receptor binding domain [13,14]. In addition, lentiviral pseudotypes (PTs) containing SARS-CoV-2 proteins have been used instead of SARS-CoV-2 virus to assess seroconversion [15]. While these PT assays provide a good correlation to ELISA, the diagnostic parameters and correlations to classical virus neutralisation assay remain largely unexplored.

ELISA offers another platform for antibody testing of serum or plasma samples for the presence of specific antibodies following infection with SARS-CoV-2 [16,17,18,19,20]. Existing SARS-CoV-2 ELISA platforms generally utilise SARS-CoV-2 spike or nucleocapsid recombinant antigens to identify the presence of binding antibodies. The sequential detection of IgM followed by IgG antibody over time post-symptom-onset (PSO) in human patients has been described, with early IgM (detected within the first week PSO) declining over time to leave a longer lasting IgG response (detected within 1–3 weeks PSO) [16,20,23,24,25]. These studies also suggest transient serological responses PSO, except perhaps for individuals with higher titres [16,20,23,24,25]. Time PSO for antibody development and detection is likewise acknowledged in the sensitivity estimates of antibody tests, suggesting higher seropositivity levels from around 2 weeks PSO [26].

The development of serological tests to detect antibodies specific to SARS-CoV-2 was originally limited by both the availability of sera with which to assess responses and, where virus neutralisation is assessed, high biological containment facilities within which to undertake live virus assays. The development of ELISA tests has likewise grown with the increased availability of serum samples, virus, and viral proteins. Here, we compared the neutralising and IgG antibody titres in COVID-19 patients at single and at multiple time points PSO using a SARS-CoV-2 VNT and two separate ELISAs, comparing the sensitivity and specificity of both methods. We also investigated the diagnostic application of these tests used individually or in combination, and we propose and validate the diagnostic application of a novel SARS-CoV-2 pVNT to provide an alternative COVID-19 functional serological assay at a lower biological containment level. These data presented herein provide information on the serological response in convalescent patients and provide a series of tools for diagnostic and research application to control SARS-CoV-2.

## 2. Materials and Methods

### 2.1. Viruses

The SARS-CoV-2 virus strain 2019-nCoV/Italy-INMI1 (clade V) was acquired from the European Virus Archive Global (EVAg) (008V-03893, www.european-virus-archive.com and used for VNT and ELISA. The Wuhan-Hu-01 spike protein sequence was used for the development of pseudotype assays. The complete sequence was submitted to GenBank (SARS-CoV-2/INMI1-Isolate/2020/Italy: MT066156) and is available on GISAID website (BetaCoV/Italy/INMI1-isl/2020: EPI_ISL_410545). For use outside of containment, the virus was inactivated using beta-propiolactone (BPL) as described previously [27].

### 2.2. Sera

APHA Control sera—Archived human health surveillance samples (*n* = 138 total) collected between January 2015 and February 2019 were used as a control cohort for this study.

APHA COVID-19 positive sera—serum samples taken from APHA staff volunteers who had experienced COVID-19-like symptoms, had isolated, and then returned to work. Permission was obtained from all contributors prior to inclusion in the study.

University Hospital Southampton Foundation NHS Trust clinical panel—A total of 128 serum samples were collected from SARS-CoV-2 PCR-positive patients following admission to University Hospital Southampton Foundation NHS Trust between 20/03/2020 and 20/5/2020 as part of the CoV-19POC study [28]. All patients gave written informed consent, or where unable to given consent, consultee assent was obtained. The trial was approved by the South Central—Hampshire A Research Ethics Committee: REC reference 20/SC/0138, on the 16 March 2020. The protocol is available at: https://eprints.soton.ac.uk/439309/2/CoV_19POC_Protocol_v2_0_eprints.pdf (accessed on 30 August 2020). The trial was prospectively registered; ISRCTN14966673, on the 18 March 2020.

### 2.3. Serum Processing

Human blood samples were centrifuged at 800× *g* for 5 min. The serum fraction was transferred to a fresh screw cap tube within an MSC and heated at 56 °C for 30 min. Serum samples were stored at 4 °C until required.

### 2.4. ELISAs

Whole Antigen ELISA—A BPL-inactivated SARS-CoV-2 antigen (iSARS-CoV-2) was produced from Vero E6 cell cultures infected with the human strain 2019-nCoV/Italy-INMI1 (008V-03893) (courtesy National Institute for Infectious Diseases “Lazzaro Spallanzani” IRCCS, Rome, Italy). BPL-treated uninfected Vero E6 cell culture extract was used as a negative control. The dilution of supernatant used to coat ELISA plates was determined in prior test runs using VNT-positive and control negative APHA serum samples. Each serum sample was tested in a 2-well test, with the control antigen well subtracted from the iSARS-CoV-2 antigen well. ELISA plates (Nunc Maxisorp) were coated with 5 µL/well of a 1/10 dilution of iSARS-CoV-2 or control antigen in carbonate coating buffer pH 9.6. Plates were incubated overnight at 4 °C and then washed in PBS/0.1%Tween-20 before loading test serum samples. Test sera were diluted 1/100 in a sample/conjugate buffer (PBS buffer containing 1% Poly Vinyl Pyrrolidone (PVP-40, Sigma-Aldrich, Merck Life Sciences, UK), 0.001 M EDTA, and 0.005% Tween-20 and 50 µL/well was added to one iSARS-CoV-2 antigen well and one control antigen well per sample. Plates were incubated for 2 h at room temperature, and after washing, 50 µL/well of Protein-A/G-HRP diluted 1:20,000 in sample/conjugate buffer was added for one hour at room temperature. Plates were washed and 100 µL/well of TMB was added for 8–10 min, the reaction was stopped by adding 100 µL/well 0.5 M H_2_SO_4_, and plates were read at 450 nm on an ELISA reader. ELISA data were analysed using ΔOD_450nm_ (iSARS-CoV-2 antigen minus the control antigen for each sample). 

The ID Screen^®^ SARS-CoV-2-N IgG Indirect ELISA (SARS-COV-2-N) was used to measure nucleocapsid protein-specific antibody. The test is validated for human samples with a given diagnostic specificity of 99.9% (95%CI: 99.6–100%) and sensitivity of 93.3% (95%CI: 78.8–98.2%) for those persons infected for more than 15 days. This test was carried out following the manufacturer’s instructions. Raw ELISA data (OD_450nm_) was used for data analysis.

### 2.5. SARS-CoV-2 Virus Neutralisation Test (VNT)

The VNT was adapted from Loeffen et al. [29], which was developed to detect antibodies against Schmallenberg virus. In the 96-well plate format, in quadruplicate, two-fold dilutions were made of the serum sample in virus growth media (Dulbecco’s modified Eagle’s media (DMEM) (Gibco) supplemented with 2% foetal calf serum and 1% Penicillin/Streptomycin/Nystatin). Then, 100 TCID_50_ of SARS-CoV-2 virus (2019-nCoV/Italy—INMI 1 (GISAID ID EPI_ISL_410545)) was added to each well. Plates were sealed with a gas permeable plate sealer and incubated at 37 °C and 5% CO_2_ for 1 h. A back titration of the input virus was performed for each aliquot used by two-fold serial dilution in virus growth media. A negative, no virus control plate was also included. After incubation, a suspension of 5 × 10^4^ Vero E6 cells was added to each well. Plates were sealed with a gas permeable plate sealer and incubated for 5 days at 37 °C and 5% CO_2_. Each well was visualised daily for cytopathic effect (CPE) under a microscope. The titre of the virus and the samples were calculated using the Spearman–Karber method and displayed as inhibitory concentration 50% (IC_50_). The limit of detection was 2.82 IC_50_ i.e., one well out of four at a specific dilution being positive for virus neutralisation. A valid test required (i) the virus back titration to be 30 to 300 TCID_50_/well, (ii) absence of neutralisation in the negative control serum, (iii) absence of CPE in the “no virus control” plate, and (iv) the positive control within 2 IC_50_.

### 2.6. Pseudotype Production

The generation and utilisation of SARS-CoV-2 pseudotypes has been described previously [30,31]. Briefly, HEK-293T cells were maintained in Dulbecco’s Modified Eagle’s media (DMEM) plus 10% foetal calf serum and penicillin and streptomycin. After a 24 h incubation, the cells were transfected with the HIV-1 gag-pol construct, pCMV-Δ8.91 [32], the firefly luciferase reporter construct pCSFLW [33], and pcDNA3-SARS2-S [34] at a ratio of 1:1.5:1 respectively, again using Fugene 6 (Promega, Madison, WI, USA). Forty-eight h later, supernatants were collected, filtered through a 0.45 μM filter, and titred on cells transfected with pCAGGS-ACE2 and pCAGGS-TMPRSS2 at a ratio of 1:3 using Fugene 6. Pseudotype particles were subsequently stored at −70 °C.

### 2.7. Pseudotype Virus Neutralisation Test (pVNT)

Twenty-four h prior to running the assay, HEK293T/17 cells were transfected with pCAGGS-ACE2 and pCAGGS-TMPRSS2 at a ratio of 1:3 using Fugene6 (Promega, Madison, WI, USA). In a 96-well plate, sera was initially diluted 1:40, and then, a 2-fold serial dilution was undertaken across the plate. Then, 100 TCID_50_ of the SARS-CoV-2 pseudotype virus that resulted in an output of 1 × 10^4^ relative light units was added to the test sera and incubated at 37 °C for 1 h before 2 × 10^4^ transfected HEK293T/17 cells were added to the sera/pseudotype virus mix. Following incubation for 48 h, the media was removed and replaced with 50 μL serum free DMEM, 50 μL of Bright Glo (Promega, Madison, WI, USA) reagent was also added at this stage. Luciferase activity was measured 2.5 min later using an Infinite 200 PRO luminometer (TECAN, Männedorf, Switzerland) plate reader. IC_50_ values were determined as described by Ferrara and Temperton [30].

## 3. Results

### 3.1. Clinical Presentation across Sampled Cohorts

Serum samples were obtained from 103 adult patients admitted to University Hospital Southampton Foundation NHS Trust displaying COVID-19 symptoms during the first wave of the pandemic in the UK, between 20 March and 30 May 2020, as part of the CoV19POC study [28]. All patients were PCR positive for SARS-CoV-2 RNA on upper respiratory tract samples using the QiaStat-Dx Respiratory SARS-CoV-2 Panel (QIAGEN, Hilden, Germany). All 103 patients had a serum sample taken on admission, 11 of these had one further sample taken on average 13 days later, and 7 had two further samples taken: one on average 11 days later and one on average 25 days later. The median (IQR) age of patients was 61 (47 to 75) years, and 62 (61%) of 103 were male. The median (IQR) duration of illness at presentation was 7 (4 to 10) days. The median (IQR) NEWS2 (National early warning score 2) was 5 (3 to 7), and 89 (90%) of 99 had evidence of pneumonia on their chest X-ray. Thirty (29%) of 103 were admitted to the intensive care unit, and 14 (14%) of 101 died within 30 days of admission. The baseline characteristics and outcomes for these patients are summarised in Table 1.

A further panel of samples were taken from individuals that had self-isolated following a positive COVID-19 test result between 1/03/20 and 1/07/20 but who displayed only mild clinical disease (cough, fever, or a loss in sense of taste or smell) and did not require hospitalisation. From these (*n* = 3), serum samples were taken intermittently for a period of 93 to 97 days post symptom onset, and antibody longevity was assessed. Furthermore, negative control human serum were retrospectively assessed from 138 samples collected prior to the emergence of SARS-CoV-2, through existing APHA staff health-surveillance schemes (samples collected between January 2015 and February 2019). These three sample sets were assessed in all three different serological assays to give a comparative readout of the performance of each assay. 

### 3.2. Serological Screening in COVID-19 Positive Patients and Symptomatic Individuals Using VNT and ELISA

**Figure 1:**
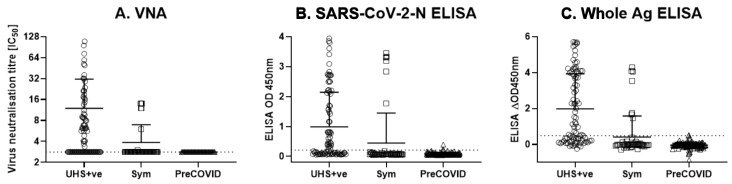
Virus neutralisation test and ELISA results from a panel of COVID-19 PCR-positive cases (*n* = 85), symptomatic individuals (*n* = 48), and healthy volunteers prior to the emergence of COVID-19 in humans (*n* = 138). (**A**) VNT; (**B**) SARS-CoV-2-N ELISA; (**C**) Whole Antigen ELISA results from (i) a group of PCR positive human patient sera from the University Hospitals Southampton NHS Foundation Trust (*n* = 85—denoted “UHS+ve”); (ii) a panel of sera from individuals that had symptomatic disease consistent with SARS-CoV-2 infection during the pandemic (*n* = 48; denoted “Sym”); and (iii) a panel of human health surveillance sera taken pre-October 2019 as a negative control group (*n* = 138; denoted “PreCOVID”). Horizontal lines represent mean +/− SD. Dotted lines represents test cut-off values of 2.83 IC50, 0.215 OD_450nm_, and 0.49 ΔOD_450nm_ for the VNT, SARS-CoV-2-N ELISA, and Whole Ag ELISA, respectively.

Serum samples from the three cohorts were tested using a VNT and two ELISAs; an in-house developed ‘Whole Antigen ELISA’ using BPL-inactivated cell culture SARS-CoV-2 virus, and a commercially available SARS-CoV-2-N ELISA (ID Screen^®^ SARS-CoV-2-N IgG Indirect ELISA), as a comparator (Figure 1A–C). The cohorts were tested using the three methods in parallel, including (i) a group of PCR positive human patient sera from the University Hospitals Southampton NHS Foundation Trust (*n* = 85—denoted “UHS+ve”); (ii) a panel of sera taken at defined time points from individuals that had symptomatic disease consistent with SARS-CoV-2 infection during the pandemic (*n* = 48; denoted “Sym”); and (iii) a panel of human health surveillance sera taken pre-October 2019 as a negative control group (*n* = 138; denoted “PreCOVID”). 

Six of the 48 serum samples from the Sym group were positive (above the limit of detection of 2.83 IC_50_) for SARS-CoV-2 neutralising antibodies in the VNT (Figure 1A). The SARS-CoV-2-N ELISA identified all six VNT-positive samples tested, while the whole antigen ELISA identified 5/5 VNT-positive Sym samples tested (there was insufficient volume to test for the 6th sample), plus two further Sym individuals that were VNT-negative (Figure 1). Of the 85 UHS+ve individuals, 45 serum samples tested positive for neutralising SARS-CoV-2 antibodies by VNT with the highest value of 109.2 and a mean IC_50_ titre of 20.1 (Figure 1A). Using ELISA on the same samples, 47 were SARS-CoV-2-N ELISA test-positive and 49 were whole antigen ELISA test-positive (Figure 1B,C). None of the 138 Pre-COVID control serum samples collected between 2015 and 2019 demonstrated any SARS-CoV-2 antibody neutralisation by VNT, but there was one positive result in each of the ELISA tests (a different individual in for each test) (Figure 1).

The diagnostic cut-off for both ELISAs was determined using receiver operating characteristic (ROC) analysis using the UHS+ve cohort (*n* = 85) as a positive control group and the PreCOVID human sera as a negative control group (*n* = 138). The ROC analysis demonstrated that the SARS-COV-2-N and whole antigen ELISAs had an equal specificity of 97.8% for both tests (Figure S1A,B). In contrast, the Whole Antigen ELISA had a slightly higher sensitivity than the SARS-CoV-2-N ELISA (57.7% compared to 55.3%, respectively) as reflected by a higher Area Under the Curve measurement for the Whole Antigen ELISA (Figure S1C). For the purpose of this analysis, test cut-offs providing a specificity of 97.8% were used, producing cut-off values of 0.215 OD_450nm_ and 0.49 ΔOD_450nm_ for the SARS-CoV-2-N ELISA and Whole Antigen ELISA respectively). 

### 3.3. Longitudinal Assessment of COVID-19 Serological Responses over Time

**Figure 2:**
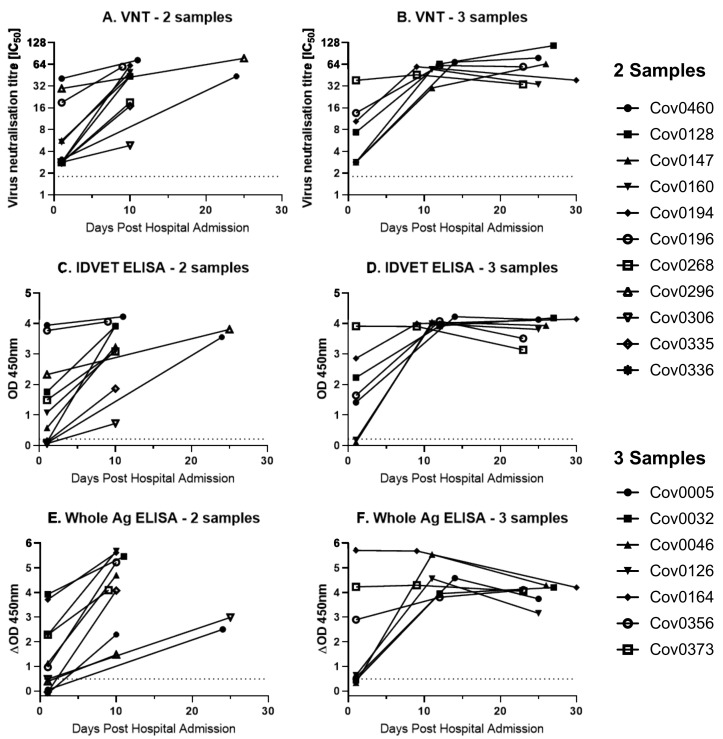
Sequential antibody testing of UHS PCR-positive individuals for which sequential samples were available. Sequential samples from a group of PCR positive human patient sera from the University Hospitals Southampton NHS Figure 2. N ELISA, and (**E**,**F**) Whole Antigen ELISA. (**A**,**C**,**E**) Eleven individuals had two sequential samples; (**B**,**D**,**F**) seven individuals had three sequential samples. Dotted line represents test cut-off values of 2.83 IC_50_, 0.215 OD_450nm_, and 0.49 ΔOD_450nm_ for the VNT, SARS-CoV-2-N ELISA, and Whole Ag ELISA, respectively.

Following this initial assessment on single time point samples, seropositivity over a longitudinal time course was investigated. VNT and both ELISAs were performed on serum samples obtained at multiple (maximum three) time points post admission from the UHS+ve cohort. Of the 11 UHS+ve individuals, for which two sequential samples were available, VNT and the Whole Antigen ELISA showed that six were positive at the first time point, compared to seven positives using SARS-CoV-2-N ELISA (Figure 2A,C,E). All UHS+ve samples were positive to all tests by the second time point. Of the seven individuals for which three sequential samples were available (Figure 2B,D,F), four were positive by VNT at the first time point, whilst five were positive by both ELISAs. All UHS+ve samples were positive on all three tests at the second and third sampling time points post admission.

**Figure 3:**
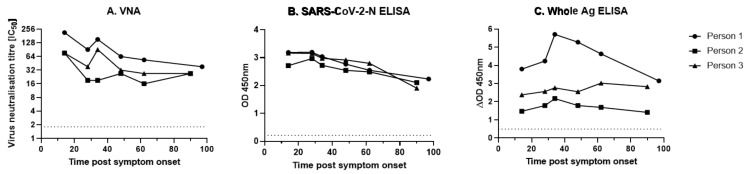
Sequential antibody testing of three VNT-positive COVID symptomatic individuals. Samples were taken for up to 93 or 97 days post symptom onset from three COVID-19 symptomatic individuals tested in Figure 1. Serum was tested using (**A**) VNT, (**B**) IDVET ELISA, and (**C**) Whole Antigen ELISA. The dotted line represents test cut-off values of 2.83 IC_50_, 0.215 OD_450nm_, and 0.49 ΔOD_450nm_ for the VNT, SARS-CoV-2-N ELISA, and Whole Ag ELISA, respectively.

Alongside the UHS+ve panel of hospital admission sera, three individuals in the Sym group that had recovered from a short course of clinical disease consistent with COVID-19 were sampled approximately every 14 days until 42 days and then monthly until 93 or 97 days. Serum samples were tested by both VNT and both ELISAs (Figure 2). The neutralising antibody levels in these three individuals declined rapidly in the first 28 days post symptom onset before plateauing or slowly declining until 93 or 97 days post infection (Figure 3A). Similarly, the SARS-CoV-2-N ELISA showed a steady decline in antibody levels for all individuals (although all remained sero-positive) from 28 days post symptom onset until 93 or 97 days post symptom onset (Figure 3B). The Whole Antigen ELISA (Figure 3C) demonstrated that for all individuals, a positive antibody response at the outset that was sustained to similar levels throughout the testing period.

### 3.4. Comparative Performance of Serological Tests across Sample Cohorts

**Figure 4:**
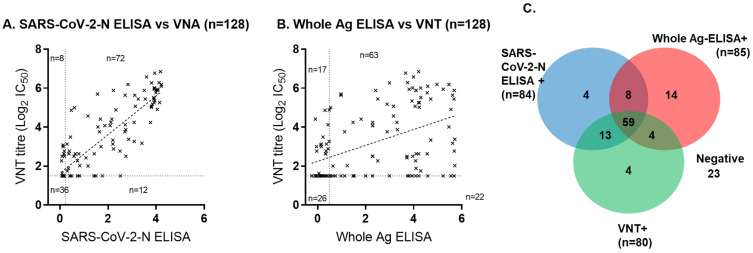
Comparison between SARS-CoV-2-N ELISA, Whole Antigen ELISA, and SARS-CoV-2 virus neutralisation test. Comparison between VNT titres and (**A**) SARS-CoV-2-N ELISA and (**B**) Whole Antigen ELISA for all 128 serum samples from 103 separate PCR positive human patients from the University Hospitals Southampton NHS Foundation Trust. (**C**) Test-positives identified by the three tests are shown in the Venn diagram.

The VNT was compared to both ELISA tests using all 128 UHS+ve samples (Figure 4). The VNT and SARS-CoV-2-N ELISA showed a strong correlation with an R^2^ of 0.7318, suggesting comparability between these methods of detection (Figure 4A). In contrast, the Whole Antigen ELISA had a poor correlation to VNT titres, producing an R^2^ of 0.2135 (Figure 4B). Overall, 80 (62.5%), 84 (65.6%), and 85 (66.4%) of the 128 UHS+ve serum samples from 103 COVID-19 confirmed patients tested positive by the VNT, SARS-COV-2-N ELISA, and Whole Antigen ELISA, respectively (Figure 4C), while 23 (18%) samples tested negative by all assays. Test-positivity to the VNT and ELISA tests is illustrated by Figure 4C.

Then, ELISA test performance was compared on the initial entry sample for each of the 103 UHS PCR-positive patients, and the ELISA results are shown in context with VNT test-positivity (Table S1). These data indicated that using a combination of VNT plus an ELISA test in parallel can increase the sensitivity of antibody detection and that the added value of the antibody test may depend upon the test used. Our data showed that VNT alone detected 53% (55 out of 103) of UHS+ve individuals, VNT plus SARS-CoV-2-N ELISA detected 65% (55 + 12 out of 103) of UHS+ve individuals, and VNT plus Whole Antigen ELISA detected 75% (55 plus 22 out of 103) of UHS+ve individuals.

### 3.5. Evaluation of a Lower Containment Level Pseudotype Assay and Comparison with VNT

**Figure 5:**
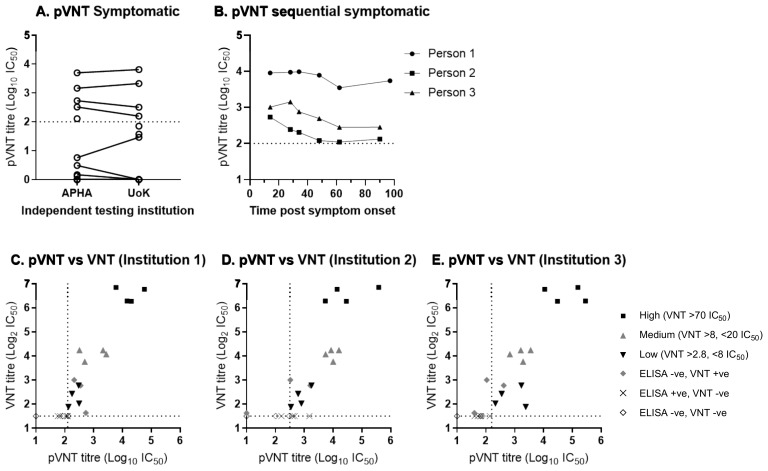
Reproducibility of pseudotype SARS-CoV-2 virus neutralisation test (pVNT) and comparison to the SARS-CoV-2 virus neutralisation test (VNT). (**A**) Titres from the pVNT, conducted and analysed independently at two separate institutions, from a subset of sera from individuals that had symptomatic disease consistent with SARS-CoV-2 infection during the pandemic tested by VNT and ELISA in Figure 1. (**B**) pVNT titres from samples taken for up to 93 or 97 days post symptom onset from three COVID-19 symptomatic individuals tested in Figure 2. (**C**–**E**) Comparison between SARS-CoV-2 VNT and pVNT on a subset serum samples from a group of PCR positive human patient sera from the University Hospitals Southampton NHS Foundation Trust. Comparison of pVNT titres on the serum panel using independently generated pseudotyped viruses from three separate locations; (**C**) APHA (institution 1), (**D**) University of Kent (Institution 2) and (**E**) University of Sussex (institution 3).

Following the evaluation of sera in a VNT using live virus, panels of sera were assessed using a lentiviral pseudotype virus expressing the SARS-CoV-2 surface glycoprotein in a pseudotype virus neutralisation test (pVNT) [34]. To determine the ability of pseudotype virus to be neutralised by SARS-CoV-2 antibody positive sera, a panel of serum from the Sym individuals, previously analysed in Figure 1, were subsequently analysed by pVNT. In the pVNT, five positive samples were identified that were also VNT positive (Figure 5A) (the sixth sample identified as positive by VNA was not analysed by pseudotype virus). To determine the robustness of the pVNT, the serum samples from the Sym group panel were also tested using the pVNT method but with an independently produced lentivirus pseudotype stock generated at the University of Kent (UoK). 

Analysis of pVNT generated and performed at the UoK determined four positive samples against SARS-CoV-2 (Figure 5A) (the fifth and sixth VNA positive sample was not analysed as samples had been depleted), demonstrating 100% diagnostic reproducibility at two independent institutions, with two independently produced batches of lentivirus pseudotypes. To determine the specificity and cross-reactivity with seasonal coronaviruses, 22 of the PreCOVID panel were analysed using pVNT. All samples gave titres of <1:40, which were classed as negative for SARS-CoV-2 antibodies (data not shown). To further confirm the use of pVNT as an alternative to VNT, the pVNT was performed on the time-course of sera from the three individuals in the Sym group, which was previously tested (Figure 3), using the pseudotype virus generated at APHA. Analysis showed that for person 1, antibody levels remained high and relatively constant throughout the time-course (Figure 5B), whilst patients 2 and 3 showed a small decrease in antibody titre until 62 and 48 days post symptoms respectively, after which antibody levels plateaued. The analysis of all three different cohorts (Sym, UHS+ve, Sym time course) have demonstrated that the pseudotype virus can be used to analyse antibody responses against SARS-CoV-2.

To further analyse the use of pVNT as an alternative to traditional VNT, a panel of serum from the UHS+ve COVID-19 confirmed patients was selected based on the titres from VNT, NP-ELISA, and Whole Antigen ELISA. The panel consisted of 23 serum samples, including those with (i) high VNT titres (>70 VNT IC_50_ [*n* = 4]), (ii) medium VNT titres (>8 and <20 [*n* = 4]), (iii) low VNT titres (>2.8 and <8 [*n* = 4]), (iv) ELISA negative and VNT positive (*n* = 3), (v) ELISA positive and VNT negative (*n* = 4), and (vi) ELISA negative and VNT negative (*n* = 4). To investigate the reproducibility of pVNT for diagnostic activities, the pVNT was analysed using a SARS-CoV-2 pseudotype virus generated at three different institutions (APHA (institution 1), The University of Kent (institution 2) and The University of Sussex (institution 3)), with all analysis carried out at APHA.

All (100%) of the high, medium, and low VNT titre sera (4/4, high; 4/4, medium; 4/4, low) were identified as containing neutralising antibody by pVNT using the pseudotype virus generated at all three institutions (Figure 5C–E). A total of 100% (4/4) of the double negative (ELISA negative and VNT negative) samples were identified as negative by the pVNT at all institutions (Figure 5C–E). The pVNT positivity of the three ELISA negative and VNT positive was quite variable; 3/3, 2/3, and 1/3 of these sera tested positive by pVNT at institutions 1, 2, and 3 respectively. Similarly, the ELISA positive and VNT negative sera exhibited a high variability in positivity by pVNT; 0/4, 3/4, and 0/4 of these sera tested positive via the pVNT. There was also a strong correlation between VNT and pVNT titres for the VNT positive sera, with R^2^ values of 0.8092, 0.7084, and 0.7405 for institutions 1, 2, and 3, respectively (data not shown). 

## 4. Discussion

Antibody detection and quantification methods are critical to assessing asymptomatic infection, post clinical convalescence, and post-vaccine responses to COVID-19. Population-based studies have given an intriguing insight into potential levels of herd immunity across asymptomatic and symptomatic individuals. However, what the outputs mean regarding population level protection is hard to define. Several population-level studies have attempted to use serological outputs to indicate the level of virus spread, both in the presence and absence of clinical disease, as well as serological titres following severe infection and convalescence. A report detailing serological responses in 61,000 individuals from Spain demonstrated that 5% of sampled individuals had antibodies specific for the spike and nucleoproteins of SARS-CoV-2 [35]. Interestingly, from this cohort, approximately 33% of sampled individuals were asymptomatic. This, as we now understand, reflects the high level of asymptomatic infection, which in turn links to the rate of transmission as well as the low case fatality rate. Neutralising antibody longevity has also been questioned, with some reports suggesting that sero-positivity wanes during convalescence, although larger studies are required. As mentioned above, some reports have suggested a correlation between clinical disease status and serological positivity; low disease severity correlating with a transient antibody response [25] and also with neutralising IgA antibodies [36]. Conversely, an Icelandic study demonstrated a high seroprevalence in those that developed clinical disease, and antibody levels were generally maintained within the first 4 months post-infection [37].

Here, we have assessed three cohorts utilising three different assays to evaluate the relevance of biological testing for antibody responses and, for a small cohort, the longevity of the antibody response. Previous studies have demonstrated that ELISA outputs can correlate quite well with neutralising antibody responses [25,36] and demonstrated high estimates of seropositivity in infected individuals, from ≈70% to 95% in some studies [16,19,25,38]. These positivity rates are dependent on time post symptom onset and also likely depend on clinical disposition and viral dose. However, ELISA has been demonstrably useful in the identification of exposed, asymptomatic contacts of SARS-CoV-2 patients, such as medical staff [39]. A high specificity also appears to be possible using ELISA platforms, from 95% to 100% [38,39]. One potentially confounding factor for antibody test specificity is that of cross-reactivity with seasonal alphacoronaviruses (NL63 and 229E) and betacoronaviruses (OC43 and HKU1). The data presented here demonstrate the utility of both live virus-based neutralisation assays as well as ELISAs that detect all binding antibodies, regardless of their role in neutralisation. For the negative control cohort, we assessed 138 serum samples taken before the emergence of SARS-CoV-2, and as such, these sera represent true negative control sera. When assessing the ability of these sera to neutralise SARS-CoV-2, none were able to neutralise the virus, suggesting that the cross reactivity of antibodies to seasonal coronaviruses are unlikely to play a role in the neutralisation of SARS-CoV-2, although there is evidence of cross neutralisation to some [40]. However, to assess this more thoroughly, either VNTs specific for seasonal coronaviruses need to be developed or specific sera raised against seasonal non-SARS-CoV-2 strains. Additionally, antibody detection by ELISA does not provide any information regarding the protection offered by antibodies. The extent to which seasonal coronaviruses may cloud serological outputs has also hampered efforts to define sero-status. Regardless, it is widely accepted that serological assays are required to define the prevalence of antibody responses across populations and any resulting humoral immunity associated with responses.

Our data illustrate the potential usefulness of applying VNT and ELISA antibody tests in parallel to enhance the sensitivity of infection detection. This enhanced sensitivity being largely due to ELISA tests identifying infected individuals before neutralising antibodies can be detected and also recognising that VNT can identify infected persons that test negative by ELISA. The level of enhanced sensitivity also depends upon the ELISA test used. We generated a relatively crude Whole Antigen ELISA using BPL-inactivated virus-infected whole Vero E6 culture extract and compared it with a commercial ELISA test, the SARS-COV-2-N ELISA kit (IDvet). Both ELISAs had an equally high specificity of 97.8% and moderate sensitivity that was broadly equivalent to that of VNT testing. The parallel VNT and ELISA testing of 103 COVID patients showed that proportionally more patients were detected using the Whole Antigen ELISA in parallel with VNT (75%), compared to the SARS-COV-2-N ELISA in parallel with VNT (65%), both ELISA tests enhancing the sensitivity of VNT alone (53%).

Interestingly, SARS-COV-2-N ELISA readouts correlated much better with VNT readouts than did the Whole Antigen ELISA. Whether this was due to a lower sensitivity of the SARS-COV-2-N ELISA that meant that SARS-COV-2-N-positives were more likely to be VNT-positive is possible; the ROC analysis suggested a lower SARS-COV-2-N sensitivity compared to the Whole Antigen ELISA, and sequential samples from COVID-19 symptomatic individuals assessed by SARS-COV-2-N ELISA also suggested a loss of sensitivity over time that was not observed using the Whole Antigen ELISA. The slightly higher sensitivity of the Whole Antigen ELISA compared to the SARS-COV-2-N ELISA may have been due to the nature of the crude antigen used, providing a broader target for polyclonal antibodies in serum samples.

The Whole Antigen ELISA approach allows for flexibility in that new virus strains can be grown and used as antigen without exactly knowing what the antigenic alterations are. This is especially important considering the emergence of new SARS-CoV-2 viral variants that may exhibit different antigenic properties. In addition, this method lends itself to comparative testing alongside seasonal and other coronavirus crude antigens. However, for a wider diagnostic use, virus cultures would need to be grown to a much higher titre than we achieved in this study, to allow for a higher antigen dilution for ELISA—and each antigen batch to go further. Recombinant antigens avoid this issue, but in our experience, both S1 and Np proteins tested, despite showing positive responses in our sequential serum samples, had overall poor sensitivity in detecting antibody-positives among the larger PCR+ cohort.

ELISA tests are relatively rapid tests compared to VNT, taking hours as opposed to days to set up and retrieve results, but ELISA results, while useful for diagnosis, may not define whether the antibody response detected is protective (neutralising), whereas VNT is recognised as a gold standard test for the positive identification of biologically active neutralising antibody. Therefore, both ELISA and VNT tests have positive credentials to bring to diagnostics.

The use of pseudotype viruses to probe the antibody responses of containment level 3 viruses has been used previously on a number of occasions [13,14,15]. Here, we examined the diagnostic and research applications of pseudotype viruses expressing the SARS-CoV-2 spike proteins as an alternative to VNT. Analysis of samples demonstrated that pseudotype viruses generated at multiple different sites were both able to detect samples previously determined positive by traditional VNT methods, with no false positives being detected; in addition, a strong correlation in antibody levels was observed when comparing pseudotype virus with traditional VNT, demonstrating a high level of sensitivity and specificity. The fact that similar results were also determined from three different SARS-CoV-2 pseudotype virus preparations demonstrates that this is a reproducible assay. Considering that resource-heavy, high containment facilities are usually needed for handling SARS-CoV-2 virus, the use of pseudotype viruses may act as a suitable alternative for modelling antibody responses in regions where these laboratory facilities are not readily available.

## Figures and Tables

**Table 1:** Baseline clinical characteristics and outcomes for patients admitted to University Hospital NHS Foundation Trust.

Characteristic	*n* = 102
Age, years	61 (47 to 75)
Male sex	62 (61)
Duration of illness, days *	7 (4 to 10)
Ct value of PCR	24 (15 to 32)
NEWS2	5 (3 to 7)
Pneumonia on CXR	89/99 (90)
Admission to ICU	30 (29)
30 day mortality	14/101 (14)

Data are presented as number (percentage) and median (interquartile range). Ct, cycle threshold; NEWS2, national early warning score 2; ICU, intensive care unit; PCR, polymerase chain reaction; * Prior to admission.

## Data Availability

All data is contained within the article or Supplementary Materials.

## Supplementary Materials

The following are available online at https://www.mdpi.com/article/10.3390/v13040713/s1, Figure S1: Receiver operating characteristic (ROC) analyses of the Whole Antigen ELISA and the IDVET ELISA., Table S1: ELISA test-positivity and VNT-positivity for the initial entry sample of 103 UHS PCR-positive COVID patients.
