# Supplementary material for: Comparison of Serological Assays for the Detection of SARS-CoV-2 Antibodies
Source: Viruses. 2021 Apr 20;13(4):713. doi: 10.3390/v13040713 (PMC8074400; doi:10.3390/v13040713)
Supplement: Supplementary file 1 [file viruses-13-00713-s001.zip › viruses-1180589-supplementary.pdf]

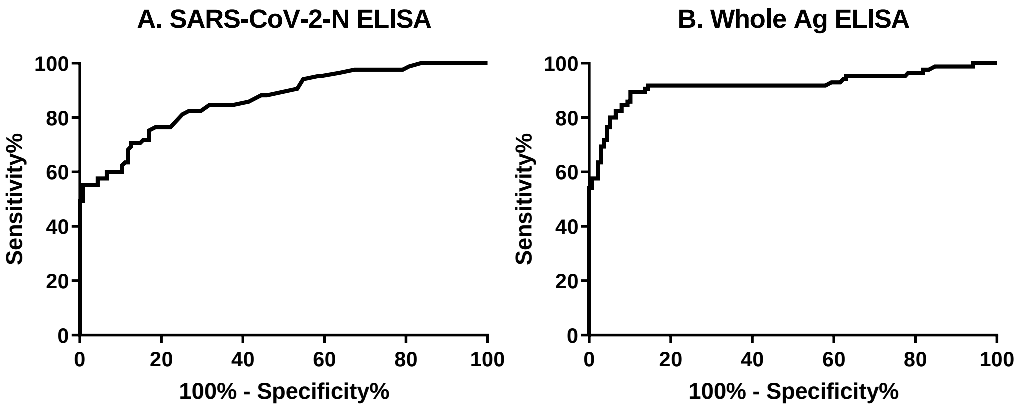

C. ROC Summary

ELISA Test	Sp, Se [95%CI] & cut-off	AUC [95%CI]<br>P value
**SARS-CoV-2-N ELISA**	Sp. 97.8% [93.6-99.5]<br>Se. 55.3% [44.1-66.1]<br>Cut-off >0.215<br>Sp. 100% [97.3-100]<br>Se. 49.4% [38.4-60.5]<br>Cut-off > 0.401	**0.8672** [0.8176-<br>P<0.0001
**Whole Antigen ELISA**	Sp. 97.8% [93.8-99.6]<br>Se. 57.7% [46.5-68.3]<br>Cut-off >0.49<br>Sp. 100% [97.4-100]<br>Se. 54.1% [43-65]	**0.9193** [0.874-0.965]<br>P<0.0001

Figure S1. Receiver operating characteristic (ROC) analyses of the Whole Antigen ELISA and the IDVET ELISA. **(A)** ROC analysis results using the single sample UoS PCR+ samples (n=85) as the positive cohort, and the APHA controls (n=138) as the negative cohort. **(C)** Area Under the Curve as a measure of test discrimination (with 0.5 representing no significant discrimination, 0.8-0.9 excellent and 0.9-1.0 outstanding)

Table S1. ELISA test-positivity and VNT-positivity for the initial entry sample of 103 UHS PCR-positive COVID patients.

	SARS-CoV-2-N ELISA		Whole Antigen ELISA	
	**Positive**	**% Positive**	**Positive**	**% Positive**
**VNT-positive (n=55)**	47	85	38	69
**VNT-negative (n=48)**	12	22	22	46
**Total (n=103)**	59	60	60	58
